# Three new species of the genus *Pseudopoda* Jäger, 2000 (Araneae, Sparassidae, Heteropodinae) from China

**DOI:** 10.3897/zookeys.1292.196539

**Published:** 2026-09-15

**Authors:** He Zhang, Feifei Cao, Lu-Yu Wang, Jie Liu, Changhao Hu

**Affiliations:** 1 College of Physics and Electronic Engineering, Xingtai University, Xingtai, Hebei, China College of Physics and Electronic Engineering, Xingtai University Xingtai China; 2 Hebei Technology Innovation Center for Digital-Intelligent Rescue Equipment, Xingtai University, Xingtai, Hebei, China Hebei Technology Innovation Center for Digital-Intelligent Rescue Equipment, Xingtai University Xingtai China; 3 Hebei Province Sweet Potato Breeding and Application Technology Innovation Center, Xingtai University, Xingtai, Hebei, China Hebei Province Sweet Potato Breeding and Application Technology Innovation Center, Xingtai University Xingtai China; 4 Arachnid Resource Centre of Hubei Province & Hubei Key Laboratory of Regional Development and Environmental Response, Faculty of Resources and Environmental Science, Hubei University, Wuhan, Hubei, China Arachnid Resource Centre of Hubei Province & Hubei Key Laboratory of Regional Development and Environmental Response, Faculty of Resources and Environmental Science, Hubei University Wuhan China; 5 Centre for Behavioral Ecology and Evolution, School of Life Sciences, Hubei University, Wuhan, Hubei, China Centre for Behavioral Ecology and Evolution, School of Life Sciences, Hubei University Wuhan China; 6 Key Laboratory of Eco-environments in Three Gorges Reservoir Region (Ministry of Education), School of Life Sciences, Southwest University, Chongqing, China Key Laboratory of Eco-environments in Three Gorges Reservoir Region (Ministry of Education), School of Life Sciences, Southwest University Chongqing China; 7 College of Life Sciences, Hunan Normal University, Changsha, Hunan, China College of Life Sciences, Hunan Normal University Changsha China

**Keywords:** Biodiversity, huntsman spiders, morphology, taxonomy

## Abstract

Three new species of the Huntsman spider genus *Pseudopoda* Jäger, 2000 (Araneae, Sparassidae) from China are described: *P. chengkouensis* Zhang, Wang, Liu & Hu, **sp. nov**. (male, female; Chongqing Municipality, China), *P. liyihangi* Zhang, Wang, Liu & Hu, **sp. nov**. (male, female; Shaanxi Province, China) and *P. maolanensis* Zhang, Wang, Liu & Hu, **sp. nov**. (male, female; Guizhou Province, China). Photos, diagnoses, and detailed descriptions of the three species are provided.

## Introduction

*Pseudopoda* Jäger, 2000 is currently the most species-rich genus of Sparassidae, comprising 271 described species, of which 168 have been recorded from China (World Spider Catalog 2026). China therefore represents the principal centre of diversity of the genus. Members of *Pseudopoda* are distributed mainly in East, South, and Southeast Asia, and many species seem to have relatively restricted distributions (Zhang et al. 2023).

Despite the remarkable diversity of *Pseudopoda* in China, the genus is still insufficiently known in this country. Following the comprehensive revision by Zhang et al. (2023), in which 99 new species were described from East, South, and Southeast Asia, additional new species from China were reported in subsequent studies in 2024 and 2025 (Chang et al. 2024; Li et al. 2024; Wen et al. 2024; Wu et al. 2024; Cao et al. 2025; Kuntner et al. 2025; Ma and Zhang 2025; Zhang et al. 2025a, 2025b, 2025c, 2025d). These continued discoveries suggest that further surveys will likely reveal more undescribed taxa in China.

During the examination of specimens collected from China, we identified three undescribed species of *Pseudopoda*. In the present study, these species are described and illustrated in detail.

## Material and methods

The specimens were preserved in absolute alcohol and examined under an OLYMPUS SZX7 stereomicroscope. Photographs were taken using Leica M205 C stereomicroscope. The male palp and the epigyne were examined and photographed after dissection. Epigynes were removed and treated in warmed 0.1 mg/ml Protease K solution before studying. All morphological measurements are in millimetres (mm) and were calculated using Leica M205 C stereomicroscope. The classes of somatic size follow Jäger (2001). Eye diameters were taken at the widest point. Palp and legs measurements were given as total length (femur, patella, tibia, metatarsus [absent in palp], tarsus). Spination follows Davies (1994). Positions of tegular appendages are given according to clock positions, based on the left palp in ventral view (Rheims and Alayón 2022). The terminology used in text and figures follows Quan et al. (2014). The specimens examined in this study are deposited in the Xingtai University (XTU, curator: He Zhang).

Abbreviations used in text and figures are grouped as follows:

Eyes and somatic structures: **ALE**, anterior lateral eyes; **AME**, anterior median eyes; **CH**, clypeus height; **DS**, dorsal shield of prosoma; **OS**, opisthosoma; **PLE**, posterior lateral eyes; **PME**, posterior median eyes.

Appendages: **Fe**, femur; **Mt**, metatarsus; **Pa**, patella; **Pp**, palp; **Ti**, tibia; **I**, **II**, **III**, **IV**, legs I–IV.

Male palp: **C**, conductor; **dRTA**, dorsal branch of RTA; **E**, embolus; **EP**, embolic projection; **RTA**, retrolateral tibial apophysis; **SD**, sperm duct; **ST**, subtegulum; **T**, tegulum; **TP**, apical tegular projection; **vRTA**, ventral branch of RTA.

Female genitalia: **AB**, anterior band; **CO**, copulatory opening; **FD**, fertilization duct; **FW**, first winding; **LL**, lateral lobe; **S**, spermatheca.

## Taxonomy

**Family Sparassidae Bertkau, 1872**

**Subfamily Heteropodinae Thorell, 1873**

**Genus *Pseudopoda* Jäger, 2000**

### Pseudopoda chengkouensis

Taxon classificationAnimaliaAraneaeSparassidae

Zhang, Wang, Liu & Hu sp. nov.

E22FE109-54F7-59A5-88D1-9E0095542AC5

https://zoobank.org/EB89318A-5BFF-430A-9006-8F038074D86F

Figs 1, 2, 3, 10

#### Type material.

***Holotype*** male: **China** • ***Chongqing Municipality***: Chengkou County, Lianfeng Village, Nacaigou, 32.0500°N, 108.7002°E, elev. 1284 m, 4 April 2025, Qianle Lu & Luyu Wang leg. (XTU, S2025090017). ***Paratypes*** • 1 male, 10 females, with same data as holotype (XTU, S2025090004–S2025090011, S2025090015–S2025090016, S2025090026); 1 male, 2 females, Chuangziping, Liaozi Township, Chengkou County, 31.7335°N, 108.7501°E, elev. 1376 m, 3 April 2025, Qianle Lu & Luyu Wang leg. (XTU, S2025090012, S2025090014, S2025090018); 1 female, Huajiaowan, Heyu Township, Chengkou County, 31.9169°N, 109.0502°E, elev. 1926 m, 5 April 2025, Qianle Lu & Luyu Wang leg. (XTU, S2025090013).

#### Etymology.

The specific name is derived from the name of the type locality.

#### Diagnosis.

**Figure 1.**
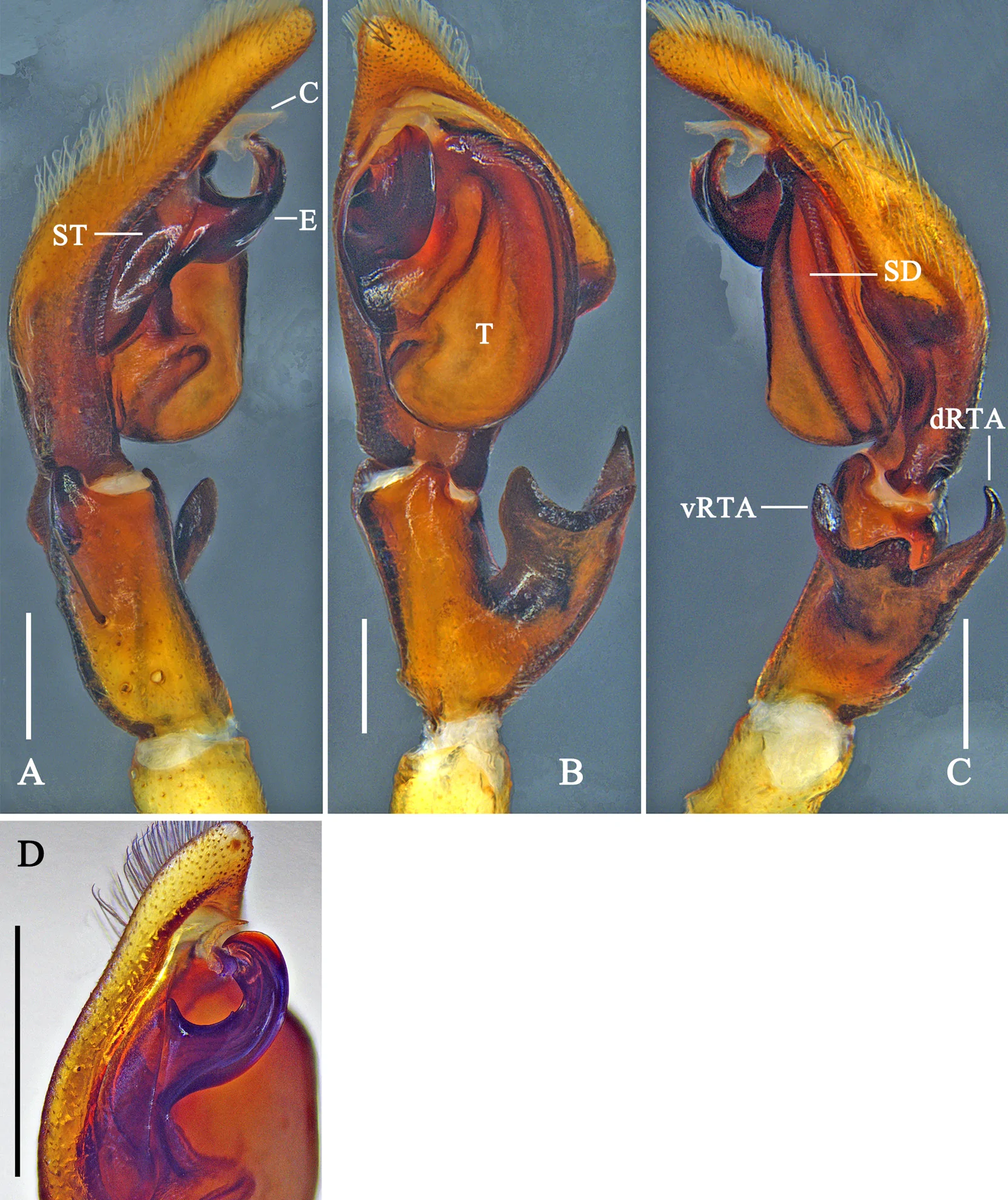
*Pseudopoda chengkouensis* Zhang, Wang, Liu & Hu, sp. nov. from Chongqing Municipality, left palp of male, holotype. **A**. Prolateral view; **B**. Ventral view; **C**. Retrolateral view. **D**. Detail of embolus, prolateral view. Abbreviations: C, conductor; dRTA, dorsal branch of retrolateral tibial apophysis; E, embolus; SD, sperm duct; ST, subtegulum; T, tegulum; vRTA, ventral branch of retrolateral tibial apophysis. Scale bars: 0.5 mm.

**Figure 2.**
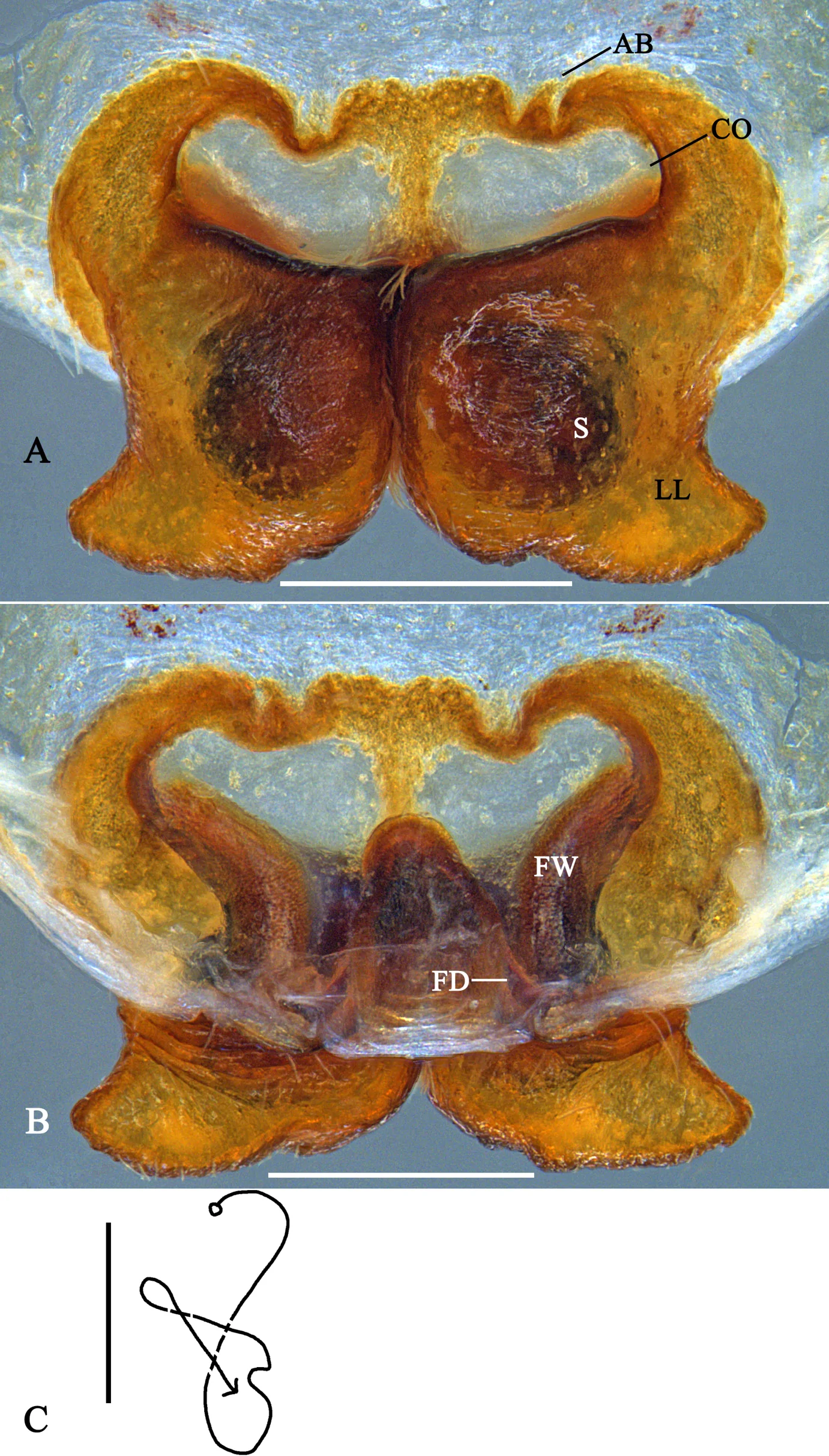
*Pseudopoda chengkouensis* Zhang, Wang, Liu & Hu, sp. nov. from Chongqing Municipality, female, paratype. **A**. Epigyne, ventral view; **B**. Vulva, dorsal view; **C**. Schematic course of internal duct system, dorsal view. Abbreviations: AB, anterior band; CO, copulatory opening; FD, fertilization duct; FW, first winding; LL, lateral lobe; S, spermatheca. Scale bars: 0.5 mm.

Male of *Pseudopoda chengkouensis* Zhang, Wang, Liu & Hu, sp. nov. resembles that of *P. yintiaoling* Deng, Zhong, Irfan & Wang, 2023 (cf. Fig. 1 and figs 33, 34, 38–40 in Deng et al. 2023) in having the RTA broad, with the ventral part with two distinct subtriangular marginal projections, but it can be distinguished from *P. yintiaoling* by the following: 1) distal part of E narrower, tapering to a pointed tip, with EP shorter, appearing as a small hump; 2) T ventrally in proximal half slightly bulged; and 3) distal part of vRTA shorter and sharper (distal part of E broad with round tip, with EP large, hook-shaped in prolateral view, T ventrally in proximal half strongly bulged, distal part of vRTA broad and round in *P. yintiaoling*). Female of *P. chengkouensis* Zhang, Wang, Liu & Hu, sp. nov. is similar to that of *P. kunmingensis* Sun & Zhang, 2012 (cf. Fig. 2 and fig. 9B–D in Zhang et al. 2017) by the following: 1) posterior part of LL with bent in an arc, smooth; and 2) S twisted; however, it can be distinguished from *P. kunmingensis* by the following: 1) anterior margins of LL curved; and 2) S nearly round, covered by LL and FW in dorsal view (anterior margins of LL almost horizontal, S oval, posterior part freely visible in dorsal view in *P. kunmingensis*).

#### Description.

Male: ***Measurements***: small-sized Heteropodinae. Body length 7.2, DS length 3.5, width 3.1, OS length 3.7, width 2.3. ***Eyes***: AME 0.16, ALE 0.25, PME 0.19, PLE 0.23, AME–AME 0.15, AME–ALE 0.08, PME–PME 0.20, PME–PLE 0.30, AME–PME 0.26, ALE–PLE 0.23, CHAME 0.34, CHALE 0.27. Chelicerae with 3 promarginal and 4 retromarginal teeth. ***Spination***: Pp: 131, 101, 2110; legs: FeI–II 323, III 322, IV 332; PaI–III 101, IV 000; TiI–II 2226, III–IV 2126; MtI–II 2024, III 3025, IV 3036. ***Measurements of palp and legs***: Pp: 5.3 (1.4, 0.8, 0.9, -, 2.2), I 13.8 (4.3, 1.4, 4.2, 2.8, 1.1), II 16.0 (4.7, 1.5, 4.9, 3.5, 1.4), III 10.7 (3.7, 1.0, 2.9, 2.2, 0.9), IV 13.0 (4.4, 0.6, 3.3, 3.4, 1.3). Leg formula: II-IV-I-III.

Palp (Fig. 1): as in diagnosis. T ventrally grooved, retrolaterally bulged, SD running submarginally along retrolateral margin of T. RTA arising basally from Ti. vRTA stout, basally broad, distally curved, and apically pointed; dRTA shorter, digitiform to triangular. C membranous, sheet-like, arising from T at 11:30 o’clock position. E rising from approximately 10 o’clock position on T.

**Figure 3.**
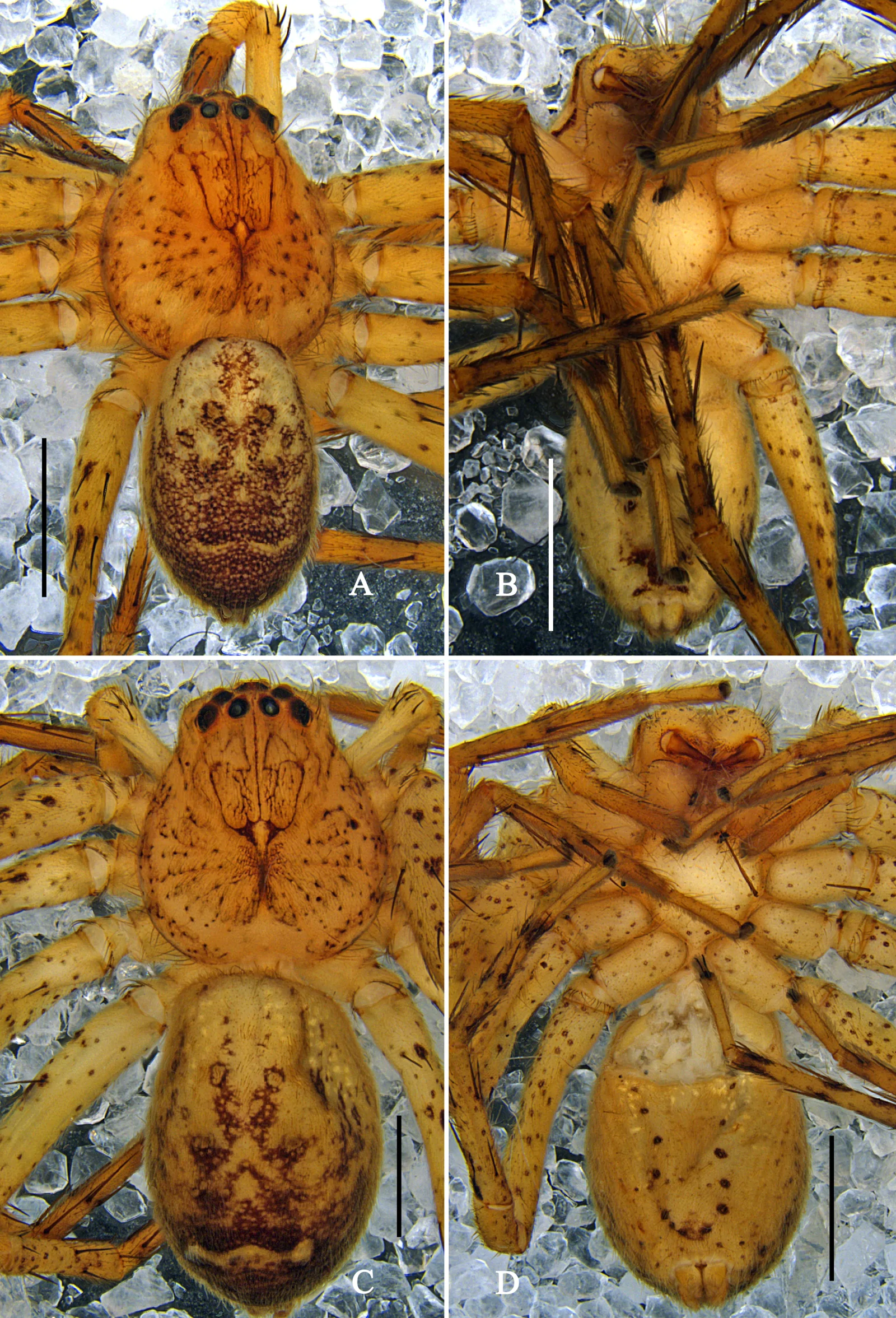
*Pseudopoda chengkouensis* Zhang, Wang, Liu & Hu, sp. nov. from Chongqing Municipality. **A**. Male habitus, dorsal view; **B**. Same, ventral view; **C**. Female habitus, dorsal view; **D**. Same, ventral view. Scale bars: 2 mm.

Colouration (Fig. 3A, B): DS yellow to orange-yellow, with dark-brown spots and distinct radial lines. Chelicerae, endites, palps, sternum, and legs yellow. Legs with scattered brown spots. OS dorsally pale yellow anteriorly, with brown to dark-brown patch medially and yellow transverse line posteriorly. OS ventrally pale yellow, with sparse, brownish spots. Spinnerets light brown.

Female: ***Measurements***: small-sized Heteropodinae. Body length 8.3, DS length 3.8, width 3.4, OS length 4.5, width 3.2. ***Eyes***: AME 0.19, ALE 0.28, PME 0.20, PLE 0.25, AME–AME 0.17, AME–ALE 0.10, PME–PME 0.24, PME–PLE 0.34, AME–PME 0.29, ALE–PLE 0.25, CHAME 0.36, CHALE 0.32. Chelicerae with 3 promarginal and 4 retromarginal teeth. ***Spination***: palp: 131, 101, 2101, 1014; legs: FeI–III 323, IV 322; PaI–IV 101; TiI–IV 2126; MtI–II 2024, III 3025, IV 3037. ***Measurements of palp and legs***: Pp: 4.9 (1.5, 0.8, 0.9, -, 1.7), I 12.3 (3.6, 1.5, 3.3, 2.9, 1.0), II 14.2 (3.8, 1.6, 3.5, 4.1, 1.2), III 10.3 (3.2, 1.3, 2.6, 2.5, 0.7), IV 11.4 (3.7, 0.9, 2.8, 3.0, 1.0). Leg formula: II-I-IV-III.

***Epigyne*** (Fig. 2): as in diagnosis. Epigynal field wider than long, with short AB. LL broad, anterior margins distinctly sclerotised, posterior parts broad. Vulva with broad FW extending anteriorly; S occupying most of posterior part of internal duct system. FD short, medially situated and posteriorly extending.

***Colouration*** (Fig. 3C, D): as in male.

#### Distribution.

Known only from the type locality (Fig. 10).

### Pseudopoda liyihangi

Taxon classificationAnimaliaAraneaeSparassidae

Zhang, Wang, Liu & Hu sp. nov.

0EB5B26E-7127-5AB4-B962-2A632EC51A17

https://zoobank.org/25348895-C080-4C82-938E-C5213B8F0A86

Figs 4, 5, 6, 10

#### Type material.

***Holotype*** male: **China** • ***Shaanxi Province***: Hanzhong City, Liuba County, Huoshaodian Town, Taiziling Village, 33.6281°N, 106.7735°E, elev. 2000–2100 m, 24 June 2025, Yihang Li leg. (XTU, S2025060001). ***Paratype*** • 1 female, with same data as holotype (XTU, S2025060002).

#### Etymology.

The specific name is derived from the name of the collector of holotype.

#### Diagnosis.

**Figure 4.**
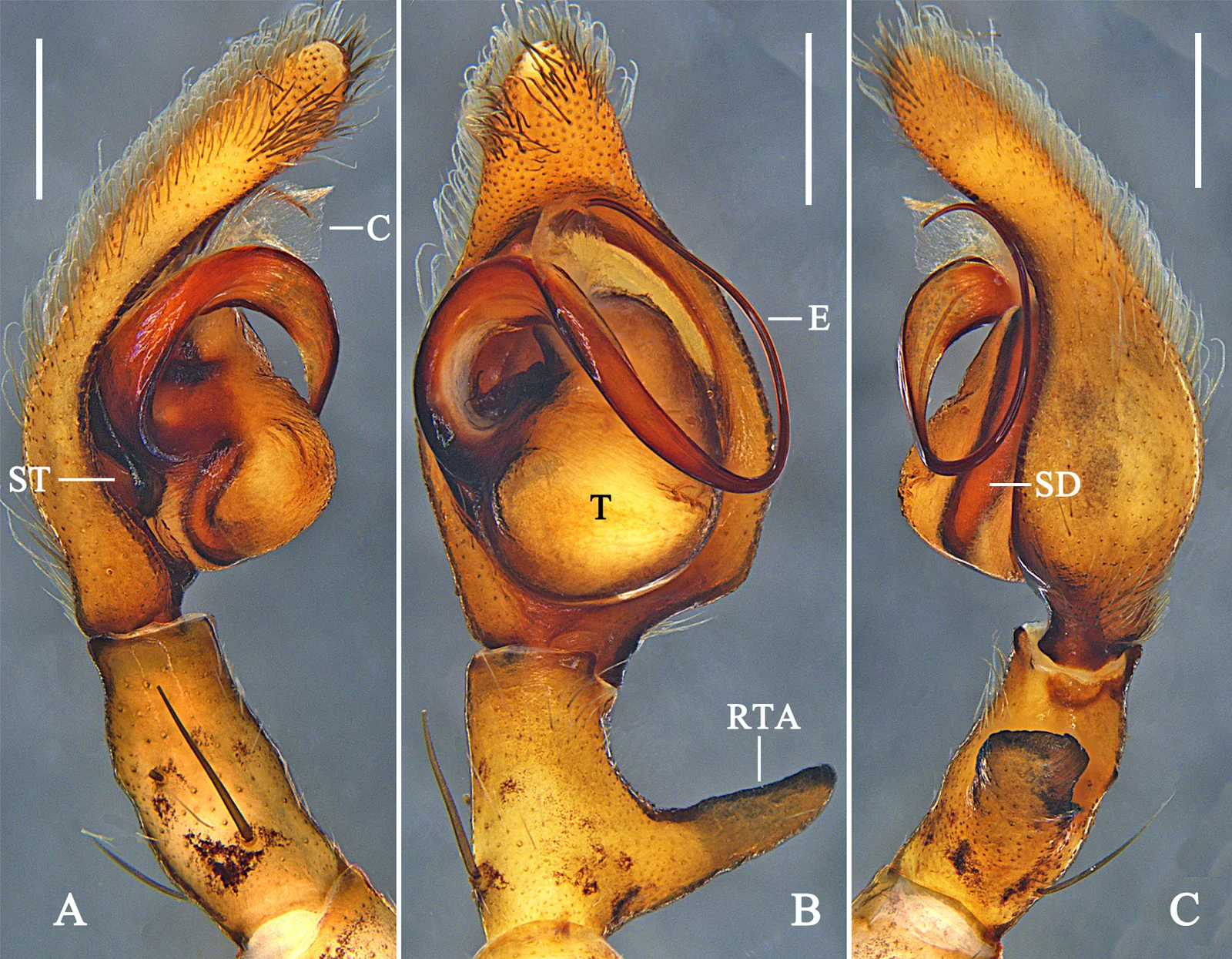
*Pseudopoda liyihangi* Zhang, Wang, Liu & Hu, sp. nov. from Shaanxi Province, left palp of male, holotype. **A**. Prolateral view; **B**. Ventral view; **C**. Retrolateral view. Abbreviations: C, conductor; E, embolus; RTA, retrolateral tibial apophysis; SD, sperm duct; ST, subtegulum; T, tegulum. Scale bars: 0.5 mm.

**Figure 5.**
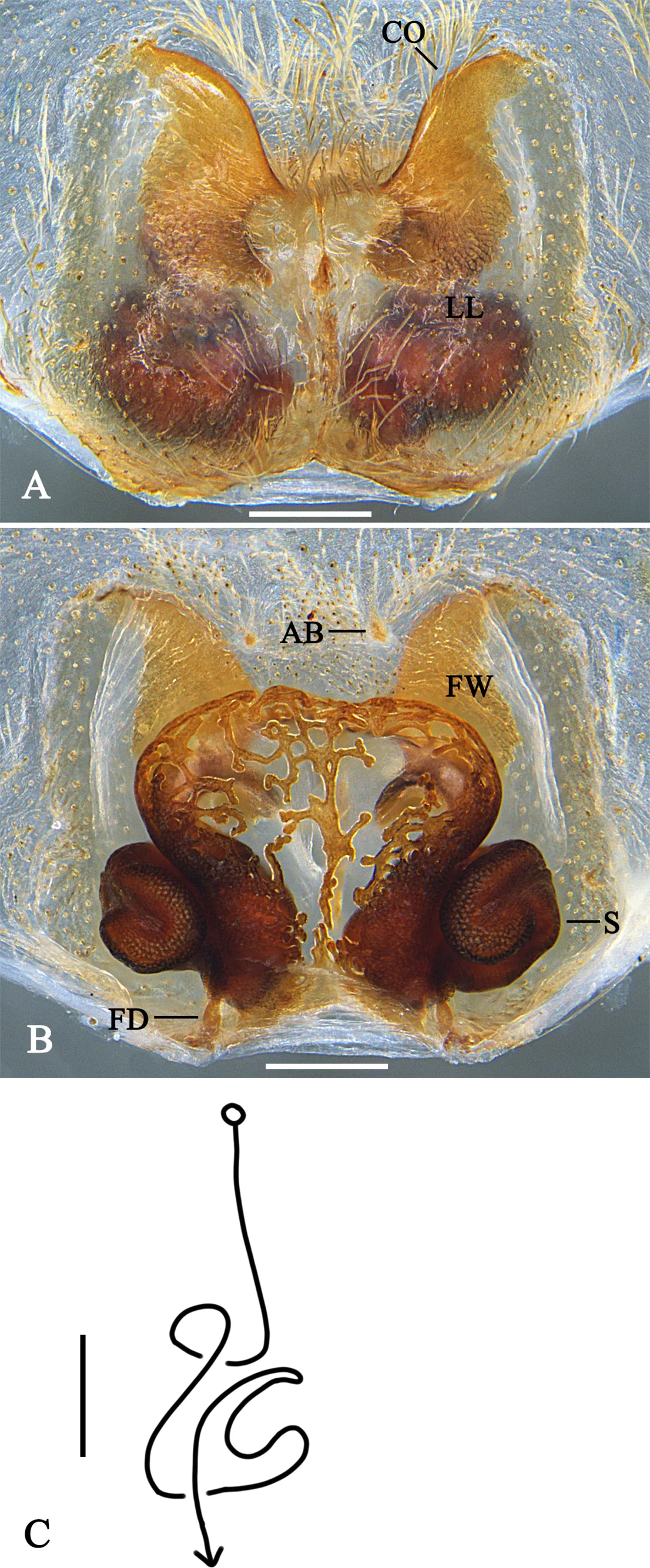
*Pseudopoda liyihangi* Zhang, Wang, Liu & Hu, sp. nov. from Shaanxi Province, female, paratype. **A**. Epigyne, ventral view; **B**. Vulva, dorsal view; **C**. Schematic course of internal duct system, dorsal view. Abbreviations: AB, anterior band; CO, copulatory opening; FD, fertilization duct; FW, first winding; LL, lateral lobe; S, spermatheca. Scale bars: 0.25 mm.

Male of *Pseudopoda liyihangi* Zhang, Wang, Liu & Hu, sp. nov. is similar to that of *P. dao* Jäger, 2001 (cf. Fig. 4 and fig. 49a–c in Jäger 2001) in having long, filiform E with distal part forming a loop, but it can be distinguished from *P. dao* by the following: 1) E arising from T at 9 o’clock position; 2) ST visible in prolateral view; 3) SD straight in retrolateral view; and 4) RTA unbranched, narrower than Ti in retrolateral view (E arising from T at 8 o’clock position; ST invisible; SD curved; RTA branched, wider than Ti in retrolateral view in *P. dao*). Female of *P. liyihangi* Zhang, Wang, Liu & Hu, sp. nov. is similar to that of *P. qingxiensis* Zhang, Jäger & Liu, 2023 (cf. Fig. 5A, B and fig. 203A, C in Zhang et al. 2023) in having reticulate part of vulva and twisted S, but it can be distinguished from *P. qingxiensis* by median part of anterior margins of LL straight (anterior margins of LL V-shaped in *P. qingxiensis*).

#### Description.

Male: ***Measurements***: small-sized Heteropodinae. Body length 8.0, DS length 4.0, width 3.6, OS length 4.0, width 3.2. ***Eyes***: AME 0.19, ALE 0.27, PME 0.17, PLE 0.25, AME–AME 0.21, AME–ALE 0.08, PME–PME 0.26, PME–PLE 0.35, AME–PME 0.23, ALE–PLE 0.29, CHAME 0.22, CHALE 0.23. Chelicerae with 3 promarginal and 4 retromarginal teeth. ***Spination***: Pp: –, 001, 3100; legs: FeI–III 323, IV 234; PaI–II 101, III 001, IV 000; TiI 3156, II–III 2126, IV 4137; MtI–II 2024, III 3025, IV 4047. ***Measurements of palp and legs***: Pp: – (–, 0.6, 0.9, -, 2.1), I 19.1 (4.9, 1.5, 5.7, 5.2, 1.8), II 20.5 (5.4, 1.6, 6.0, 5.5, 2.0), III 15.5 (4.6, 1.3, 4.1, 4.0, 1.5), IV 17.0 (4.8, 1.0, 4.7, 4.7, 1.8). Leg formula: II-I-IV-III.

Palp (Fig. 4): as in diagnosis. T kidney-shaped. RTA simple, arising from basal part of Ti. C membranous, arising from T at 12 o’clock position. E projection absent, basal part wider than 8× width of embolic tip, and gradually thinner until bottom part of embolic distal loop.

**Figure 6.**
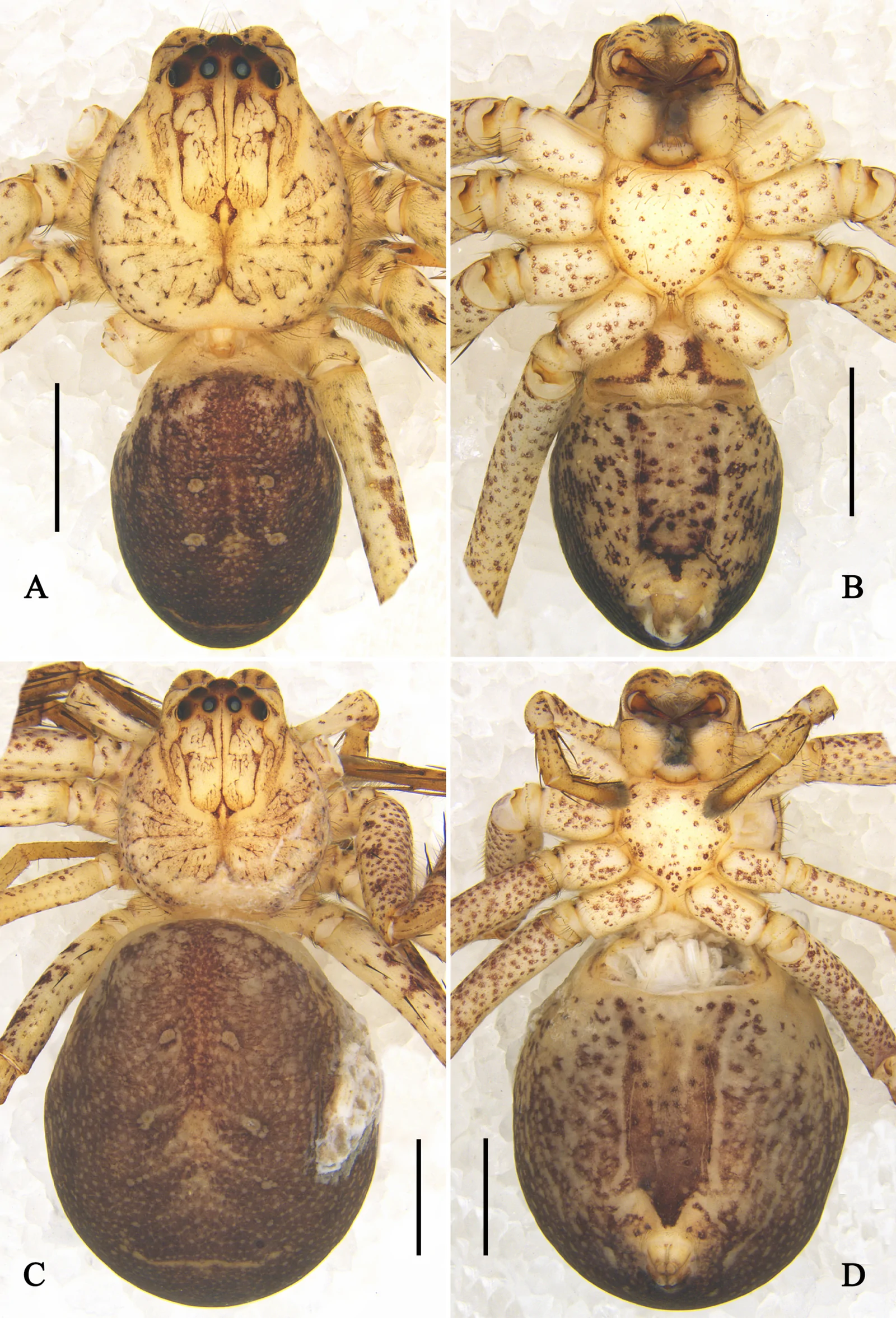
*Pseudopoda liyihangi* Zhang, Wang, Liu & Hu, sp. nov. from Shaanxi Province. **A**. Male habitus, dorsal view; **B**. Same, ventral view; **C**. Female habitus, dorsal view; **D**. Same, ventral view. Scale bars: 2 mm.

Colouration (Fig. 6A, B): DS yellow, with black spots and radial lines. Chelicerae, endites, palps, sternum, and legs yellow, with brownish-purple spots. OS dorsally brownish purple, medially with 2 pairs of brown spots and posteriorly with yellow horizontal line. OS ventrally light brown, with brownish-purple spots. Spinnerets light brown.

Female: ***Measurements***: medium-sized Heteropodinae. Body length 11.1, DS length 4.2, width 3.7, OS length 6.9, width 5.8. ***Eyes***: AME 0.17, ALE 0.25, PME 0.16, PLE 0.24, AME–AME 0.24, AME–ALE 0.09, PME–PME 0.28, PME–PLE 0.40, AME–PME 0.24, ALE–PLE 0.32, CHAME 0.37, CHALE 0.33. Chelicerae with 3 promarginal and 4 retromarginal teeth. ***Spination***: palp: 131, 101, 3120, 3020; legs: FeI–III 323, IV 331; PaI 000, II 101, III–IV 000; TiI–II 3136, III–IV 2126; MtI–II 2024, III 3024, IV 4045. ***Measurements of palp and legs***: Pp: 4.5 (1.4, 0.6, 1.0, -, 1.5), I 12.5 (3.5, 1.3, 3.5, 3.0, 1.2), II 13.0 (3.7, 1.4, 3.6, 3.1, 1.2), III 10.4 (3.2, 1.1, 2.7, 2.3, 1.1), IV 12.3 (3.8, 1.2, 3.1, 3.0, 1.2). Leg formula: II-I-IV-III.

***Epigyne*** (Fig. 5): as in diagnosis. Epigynal field slightly wider than long. AB short. Anterior margins of LL curved, more sclerotised than the rest; posterior margins wide W-shaped. Vulva medially with heart-shaped, reticulate part. Internal duct system medially with a circle, posterior S twisted. FD posteriorly situated.

***Colouration*** (Fig. 6C, D): as in male, but OS dorsally and ventrally lighter.

#### Distribution.

Known only from the type locality (Fig. 10).

### Pseudopoda maolanensis

Taxon classificationAnimaliaAraneaeSparassidae

Zhang, Wang, Liu & Hu sp. nov.

AE467947-AAAE-5967-898F-57832D21E3C4

https://zoobank.org/5F84E8E1-DDA6-4305-873C-596F21EF362C

Figs 7, 8, 9, 10

#### Type material.

***Holotype*** male: **China** • ***Guizhou Province***: Qiannan Buyi and Miao Autonomous Prefecture, Libo County, Maolan National Nature Reserve, 25.3502°N, 107.9168°E, elev. 560 m, 19 July 2025, Hailun Chen leg. (XTU, S2025070001). ***Paratypes*** • 1 male, 1 female, with same data as holotype (XTU, S2025070002–S2025070003).

#### Etymology.

The specific name is derived from the name of the type locality.

#### Diagnosis.

**Figure 7.**
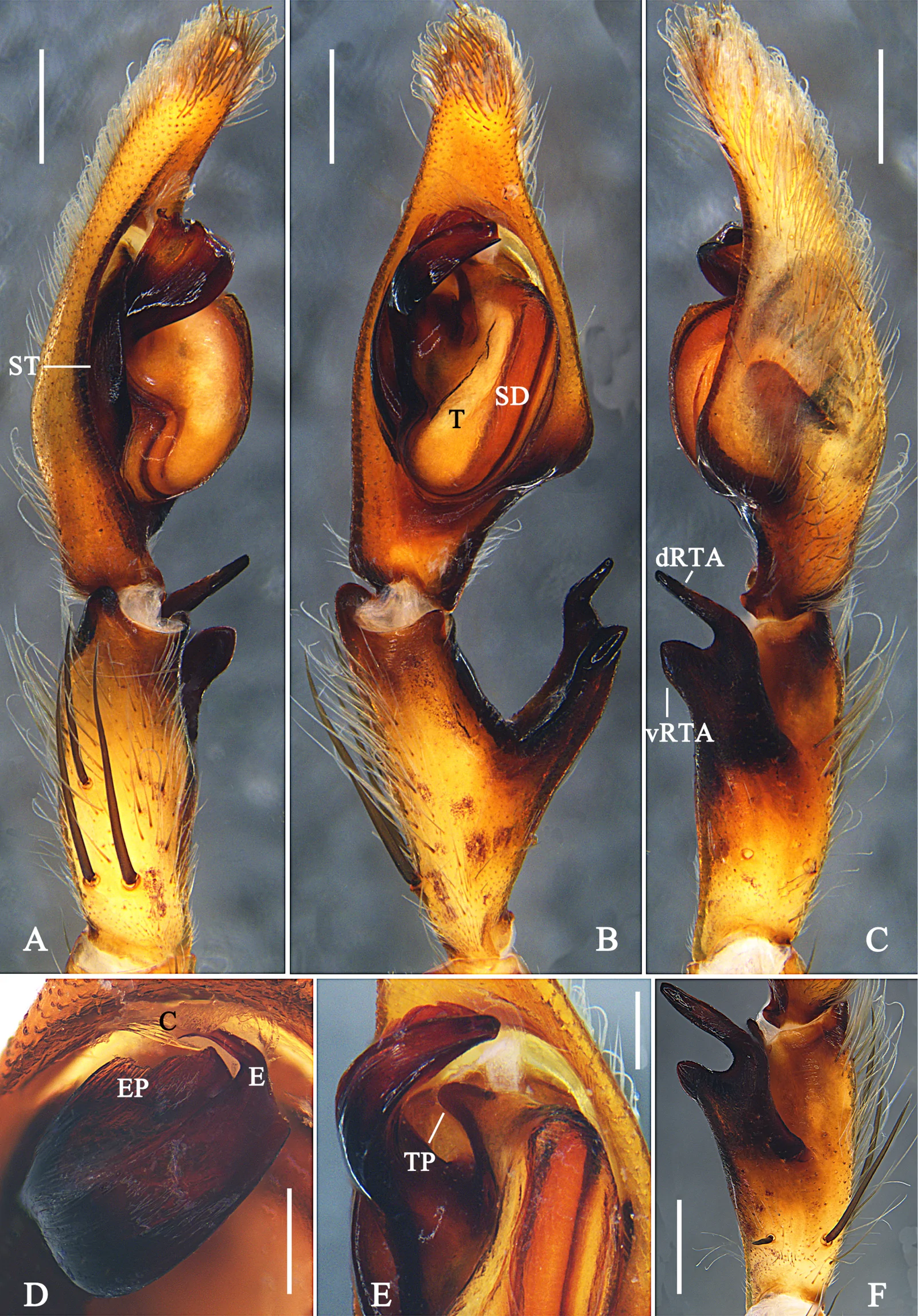
*Pseudopoda maolanensis* Zhang, Wang, Liu & Hu, sp. nov. from Guizhou Province, left palp of male. **A**. Prolateral view; **B**. Ventral view; **C**. Retrolateral view; **D**. Detail of embolic tip, ventral view; **E**. Detail of tegulum, ventral-retrolateral view; **F**. Tibia, retrolateral (**A–D** holotype **E, F** paratype). Abbreviations: C, conductor; dRTA, dorsal branch of retrolateral tibial apophysis; E, embolus; EP, embolic projection; SD, sperm duct; ST, subtegulum; T, tegulum; TP, apical tegular projection; vRTA, ventral branch of retrolateral tibial apophysis. Scale bars: 0.5 mm (**A–C, F**); 0.2 mm (**D, E**).

**Figure 8.**
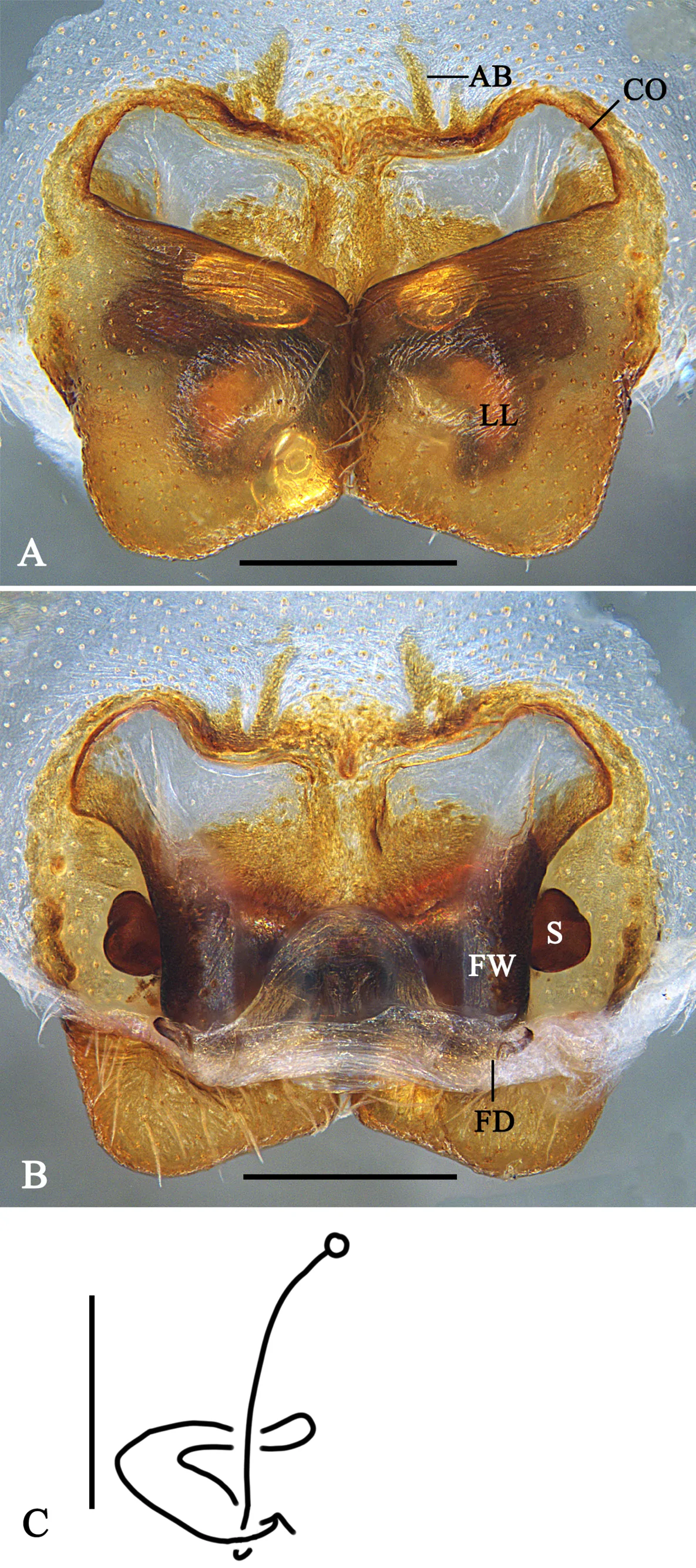
*Pseudopoda maolanensis* Zhang, Wang, Liu & Hu, sp. nov. from Guizhou Province, female, paratype. **A**. Epigyne, ventral view; **B**. Vulva, dorsal view; **C**. Schematic course of internal duct system, dorsal view. Abbreviations: AB, anterior band; CO, copulatory opening; FD, fertilization duct; FW, first winding; LL, lateral lobe; S, spermatheca. Scale bars: 0.5 mm.

Male of *Pseudopoda maolanensis* Zhang, Wang, Liu & Hu, sp. nov. is similar to that of *P. titan* Zhao & Li, 2018 (cf. Fig. 7A–E and figs 28, 29A, B in Jiang et al. 2018) in having digitiform dRTA, broad embolic base, and embolic tip bend almost 90° in ventral view, but it can be distinguished from *P. titan* by the following: 1) EP with irregular margin; and 2) RTA arising from median part of Ti, vRTA almost 1.5× wider than dRTA (EP triangular; RTA arising from basal part of Ti, vRTA almost 3.5× wider than RTA in *P. titan*). Female of *P. maolanensis* Zhang, Wang, Liu & Hu, sp. nov. is similar to that of *P. rufosulphurea* Jäger, 2001 (cf. Fig. 8A, B and fig. 50j, k in Jäger 2001) in having laterally uncovering S in dorsal view, but it can be distinguished from *P. rufosulphurea* by the following: 1) posterior part of LL almost parallel to anterior part, LL touching each other; and 2) anterior part of FW wider than posterior part (LL separated, posterior margins almost parallel to anterior margins; anterior part of FW narrower than posterior part in *P. rufosulphurea*).

#### Description.

Male: ***Measurements***: small-sized Heteropodinae. Body length 9.9, DS length 4.8, width 4.5, OS length 5.1, width 3.4. ***Eyes***: AME 0.23, ALE 0.25, PME 0.23, PLE 0.27, AME–AME 0.14, AME–ALE 0.04, PME–PME 0.34, PME–PLE 0.42, AME–PME 0.22, ALE–PLE 0.22, CHAME 0.26, CHALE 0.27. Chelicerae with 3 promarginal and 4 retromarginal teeth. ***Spination***: Pp: 131, 101, 1130; legs: FeI–IV 323; PaI–IV 101; TiI 2226, II 2326, III 2125, IV 2126; MtI 1014, II 2014, III 2024, IV 3234. ***Measurements of palp and legs***: Pp: 7.9 (2.8, 0.9, 1.4, -, 2.8), I 30.5 (8.1, 2.1, 9.2, 8.3, 2.8), II 31.8 (8.6, 1.9, 9.7, 8.8, 2.8), III 22.7 (6.8, 1.7, 6.7, 5.8, 1.7), IV 26.2 (7.7, 1.6, 7.1, 7.5, 2.3). Leg formula: II-I-IV-III.

Palp (Fig. 7A–E): as in diagnosis. Apical part of T with a projection. RTA branched, dRTA almost 1.5× longer than vRTA. Prolateral distal Ti with a small projection. C membranous, arising from T at 12 o’clock position. E arising from T at 9:30 o’clock position, basal part of E broad, medially with an edge, EP irregular.

**Figure 9.**
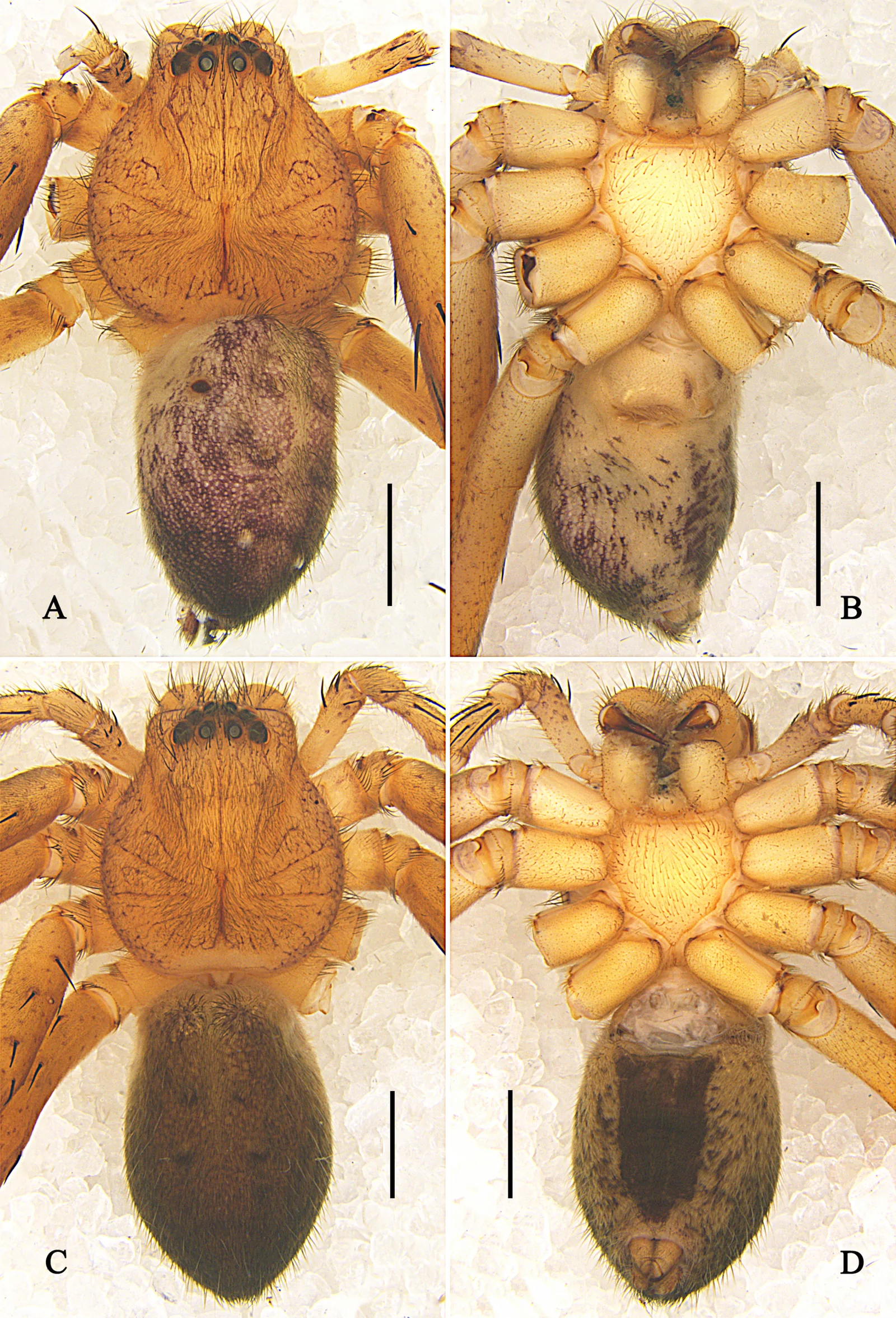
*Pseudopoda maolanensis* Zhang, Wang, Liu & Hu, sp. nov. from Guizhou Province. **A**. Male habitus, dorsal view; **B**. Same, ventral view; **C**. Female habitus, dorsal view; **D**. Same, ventral view. Scale bars: 2 mm.

Colouration (Fig. 9A, B): DS orange, with brown radial lines. Chelicerae yellow, with brown lines. Endites and sternum pale yellow. Palps and legs yellow, with brown spots. Dorsal part of OS brownish purple, anteriorly with yellowish-brown longitudinal line and posterior part with two yellow spots, venter yellowish brown, with brownish purple spots. Spinnerets yellowish brown.

Female: ***Measurements***: medium-sized Heteropodinae. Body length 10.8, DS length 5.0, width 4.6, OS length 5.8, width 3.9. ***Eyes***: AME 0.23, ALE 0.30, PME 0.23, PLE 0.29, AME–AME 0.20, AME–ALE 0.12, PME–PME 0.30, PME–PLE 0.37, AME–PME 0.28, ALE–PLE 0.25, CHAME 0.26, CHALE 0.24. Chelicerae with 3 promarginal and 4 retromarginal teeth. ***Spination***: Pp: 131, 101, 3120, 3030; legs: FeI–III 323, IV 321; PaI–IV 101; TiI 2126, II 2226, III 2125, IV 2126; MtI 2014, II 1014, III 2024, IV 3037. ***Measurements of palp and legs***: Pp: 6.7 (1.9, 0.6, 1.7, -, 2.5), I 23.6 (6.4, 2.0, 7.0, 6.2, 2.0), II 24.3 (6.9, 1.7, 7.2, 6.2, 2.3), III 18.3 (5.5, 1.5, 5.0, 4.7, 1.6), IV 21.9 (6.8, 1.8, 5.7, 5.7, 1.9). Leg formula: II-I-IV-III.

***Epigyne*** (Fig. 8): as in diagnosis. Epigynal field slightly wider than long. AB obvious. Anterior margins of LL wide V-shaped, posterior margins widely W-shaped. FW V-shaped. Interdistance between S almost as wide as interdistance between CO. FD posteriorly situated.

***Colouration*** (Fig. 9C, D): as in male, but generally darker. OS dorsally brown, with two pairs of black spots. OS ventrally with shield-shaped, dark-brown medial marking.

#### Variation.

Palpal dRTA of paratype male almost as wide as vRTA, and almost 2× longer than vRTA (Fig. 7F).

#### Distribution.

**Figure 10.**
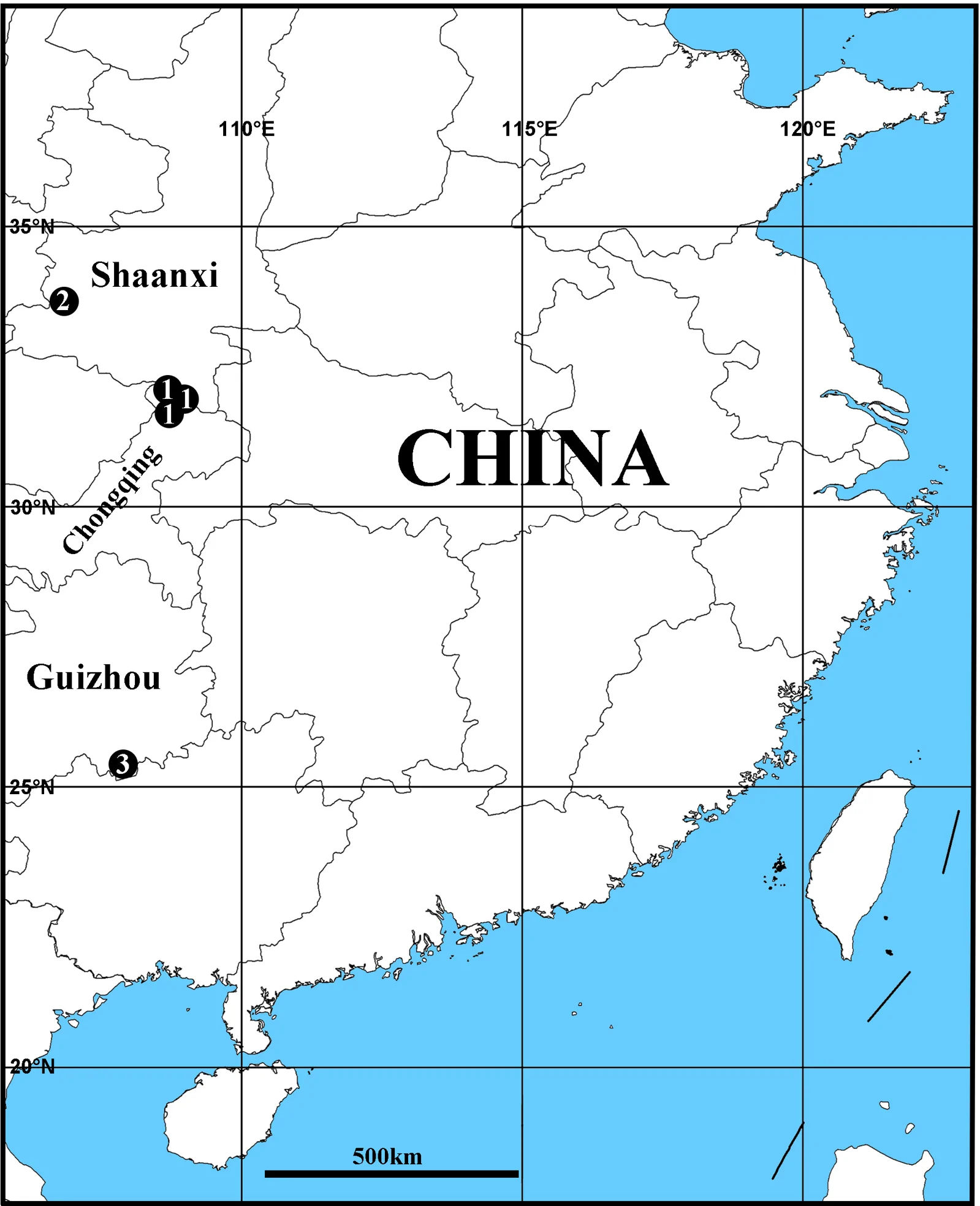
Distribution map of the three *Pseudopoda* species described in this paper. The numbers represent the different species. 1: *P. chengkouensis* Zhang, Wang, Liu & Hu, sp. nov.; 2: *P. liyihangi* Zhang, Wang, Liu & Hu, sp. nov.; 3: *P. maolanensis* Zhang, Wang, Liu & Hu, sp. nov.

Known only from the type locality (Fig. 10).

## Supplementary Material

XML Treatment for Pseudopoda chengkouensis

XML Treatment for Pseudopoda liyihangi

XML Treatment for Pseudopoda maolanensis

## Additional information

### Artificial Intelligence (AI) use

The authors accept full responsibility for the content of the manuscript, including the disclosure of any use of AI.

No AI tools were used in the preparation of this manuscript.

### Author ORCIDs

H. Zhang https://orcid.org/0000-0002-1478-9837

L.-Y. Wang https://orcid.org/0000-0002-5250-3473

J. Liu https://orcid.org/0000-0001-7744-9744

C. Hu https://orcid.org/0009-0007-5591-3121

### Data availability

All of the data that support the findings of this study are available in the main text.
